# Development of an animal component free production process for Sabin inactivated polio vaccine

**DOI:** 10.1016/j.jvacx.2022.100223

**Published:** 2022-09-30

**Authors:** Diego A. Suarez-Zuluaga, Leo A. van der Pol, Aart G. van 't Oever, Wilfried A.M. Bakker, Yvonne E. Thomassen

**Affiliations:** Intravacc, P.O. Box 450, 3720 AL Bilthoven, the Netherlands

**Keywords:** Vero cells, Design of experiments, sIPV, IPV, Eradication, Animal component free

## Abstract

Inactivated polio vaccine production using attenuated Sabin strains (sIPV) instead of wild type polio viruses (cIPV) is an initiative encouraged by the World Health Organization. This use of attenuated viruses is preferred as it reduces risks related to potential outbreaks during IPV production. Previously, an sIPV production process was set up based on the cIPV production process. Optimizing this process while using only animal component free (ACF) substances allows reduction of operational costs and mitigates risks of adverse effects related with animal derived compounds. Here, development of a process for production of sIPV using only ACF compounds, is described.

The upstream process required a change in cell growth medium from serum-containing medium to ACF medium, while virus production media remained the same as the already used M199 medium was free of animal components. In the downstream process multiple modifications in existing unit operations were made including addition of a diafiltration step prior to inactivation. After optimizing each unit operation, robustness of the whole process was demonstrated using design of experiments (DoE) methodology. By using DoE we were able to vary different process parameters across unit operations to assess the impact on our quality attributes. The developed process was robust as the observed variation for quality attributes due to differences in process parameters remained within specification.

The resulting pilot process showed not only to be robust, but also to have a considerable higher product yield when compared to the serum containing sIPV process. Product yields are now comparable to the cIPV process based on using wild type polio viruses. Moreover, the potency of the produced vaccine was comparable that of cIPV vaccine. The developed ACF sIPV process can be transferred to vaccine manufacturers at the end-of pre-clinical development phase, at lab- or pilot scale, before production of clinical trial material.

## Introduction

Poliovirus (PV) caused a considerable disease burden until the 1980s, making it one of the priorities for vaccine development. Two classical vaccine products against infectious diseases – an inactivated whole virus and a live attenuated vaccine were developed for the three virus types (1, 2 and 3) in the 1950s [1].

Jonas Salk developed an inactivated polio vaccine (IPV), using selected wild type PV strains representing the three types [1]. The process to produce this conventional IPV (cIPV) is safe and effective but also relatively expensive when compared to the manufacturing process for a live attenuated vaccine. Moreover, it does not limit transmission of PV as it does not provide mucosal immunity [2].

Albert Sabin, generated three modified PV strains for a live and attenuated PV vaccine that evokes systemic and mucosal immunity after - fast and easy - oral administration. The manufacturing of this vaccine is relatively simple and allows to produce a high amount of doses, thus supporting a low production cost. This represented an ideal vaccine. Unfortunately, modified Sabin strains can, at low frequency, revert to virulent PV, indicated as Vaccine Derived Polio Virus (VDPV). This reduced safety profile makes this oral PV vaccine (OPV) non-compatible for use after the planned eradication of PV [3].

Use of cIPV and OPV resulted in a major reduction of poliomyelitis worldwide (1960–1990), with a remaining disease burden in Low and Middle Income Countries (LMIC) [4]. Since PV has humans as only host, it seemed to provide a suitable target for the next eradication program, after successful eradication of poxvirus in 1979 [5]. Therefore, the World Health Organization (WHO) started a global PV eradication program in 1988.

The eradication program has resulted in an enormous reduction of polio cases in developing countries from 300,000 to 400,000 cases per year at the start of the Global Polio Eradication Initiative (GPEI) to less than 50 annual cases in 2016–2018, with endemic PV remaining only in two countries, Pakistan and Afghanistan [4]. Two out of three PV types have been eradicated. PV type 2 was declared eradicated in September 2015 and type 3 in October 2019. The last phase of the eradication program shows to be challenging. PV type 1 prevalence showed an increase in 2019 and 2020 [6]. In addition, occurrence of VDPV is higher than expected, in particular for PV type 2. Moreover, PV type 2 has more opportunity to spread, since vaccination against it is reduced due to the switch from trivalent to bivalent OPV (bOPV) in 2015. Therefore, bOPV vaccination with bOPV containing types 1 and 3 was maintained after eradication of PV type 3 [4].

For the eradication program, both cIPV and OPV vaccines were required. However, for an effective final phase it would be beneficial to have improved versions of these vaccines. This is safer and more cost effective for application in LMIC. An IPV using Sabin strains (sIPV) is safer to produce because of use of attenuated poliovirus.The manufacturing in LMIC countries would facilitate a lower costs price for this sIPV if a production process for sIPV with comparable efficiency to the cIPV process could be developed for Technology Transfer [7]. Development of an ACF sIPV process that meets these requirements is described here.

In the 1990s two manufacturers in Asia started in-house development of an sIPV vaccine, resulting in regulatory approval of sIPV from the Japan Poliomyelitis Research Institute (JPRI) in 2012 and from the Institute of Medical Biology, Kunming, China in 2015 [8], [9]. Because a further increase of sIPV production capacity seemed required to meet GPEI targets, WHO coordinated the development and technology transfer of a sIPV process.

An initial process, based on cIPV production, was developed for sIPV previously [3], [10]. Comparable to the cIPV process, the expansion phase of Vero cells was performed in serum-containing (SC) medium, while production of poliovirus was done in M199 medium, which is free of components of animal source. Poliovirus was purified by clarification, concentration, size-exclusion and anion-exchange chromatography prior to chmical inactivation with formaldehyde [11]. A targeted approach was chosen for process development for sIPV [10]. This assumed that limited optimization of specific process steps would be sufficient to yield a feasible production process. However, this assumption turned out to be false. In particular for PV type 2 initial process yields were low. This was mainly caused by the different isoelectric point of this virus which resulted in self-aggregation [12]. Instead of further process optimization of this base case sIPV process, it was decided to first test whether sIPV vaccine product had a comparable safety and efficacy as the cIPV. Clinical phase I & IIa studies showed that sIPV vaccine was as effective (comparable efficacy) and safe as cIPV product [13], [14], [15].

Several partners received this base case sIPV process in a Technology Transfer (TT) program for further development towards the market. As a result of this TT program, the Korean company LG Life Sciences received WHO Pre-Qualification [16] for their sIPV vaccine product (Eupolio) and the Chinese company Sinovac has approval for sIPV from their national regulatory agency [17]. Other partners are close to product approval by local Regulatory Authorities [18], [19].

While development work to improve the production process for the PV type 2 sIPV component was ongoing, testing of animal-component-free (ACF) media showed promising results in various culture modes [20]. Therefore, efforts to further optimize the sIPV process were combined with the objective to develop an ACF sIPV production process. Here, development work to obtain a feasible ACF sIPV process is described. This work focused on PV type that required most optimization (PV type 2). Since the inactivation procedure and final formulation were maintained as standard for IPV, these final steps were not addressed.

To make the target sIPV process completely free of animal components, besides a change in culture medium from serum-containing to an ACF medium, the porcine trypsin used for detachment of adherent Vero cells and for cleaning of the ultrafiltration (UF) membranes in the purification process had to be replaced by ACF variants. VP-SFM was selected as ACF medium (based upon previous experiments; data not shown). TripLE Select was chosen as replacement for cell detachment. Sodiumhydroxide was chosen as alternative for UF cleaning. The approach was to first check initial feasibility of a target ACF process by testing ACF alternatives in further similar process settings as the base case sIPV process in non-ACF conditions. Then settings for the critical process parameters were screened, explored and confirmed for different unit process steps by DoE.

The QbD approach is enabled by the d-antigen ELISA assay as reliable predictor of potency and efficacy of PV vaccine product, while assays for host cell DNA and host cell protein indicate the main contaminants of the impurity profile related to product safety. The robustness of the ACF sIPV process with initial feasibility as generated for PV type 2 was evaluated based on risk assessment to indicate relevant parameters and ranges to be tested. With this standardized pilot ACF process, sIPV vaccine product was produced for all three PV subtypes to check product quality.

## Materials and methods

### Cell and virus culture

Vero cells obtained from WHO (10–87) originally derived from ATCC (CCL-81) were used in this study. A vial from a SC-medium cell bank was thawed and diluted into VP-SFM culture medium (Life Technologies, Carlsbad, California, United States) for cell expansion. Maximum four passages were performed before adding cells to bioreactors. Each passage included transfer of Vero cells from *T*-flasks, after enzymatic detachment. This procedure consisted of rinsing twice with PBS (- Ca^2+^), detaching cells with TrypLE Select (Life Technologies, Carlsbad, California, United States), and collecting cells with a centrifugation step. The cell pellet was subsequently diluted into fresh medium and used for expansion preculture. Once a sufficient target amount of viable cells was obtained, Vero cells were added to bioreactors for growth using 3 g/L Cytodex 1 microcarriers (GE Healthcare, Chicago, Illinois, United States).

Bioreactor operation was previously described in Suarez-Zuluaga et al. (2019) [21]. Different lab-scale bioreactors were used (Applikon, Delft, The Netherlands or Sartorius, Göttingen, Germany). Working volume ranged from 2.3L to 16L. Bioreactors were run in batch mode, but glucose and glutamine were added by bolus feeding to 10 mM glucose and 2 mM glutamine when concentrations were below 5 mM and 0.5 mM respectively. During the cell growth phase, temperature was set at 37 °C, Dissolved Oxygen (DO) at 50 %, and pH at 7.2. At the selected time of infection (TOI) and prior to virus addition, media was removed and exchanged for M199 (Life Technologies, Carlsbad, California, United States). In addition, pH was switched to 7.4, DO was reduced to 25 %, and temperature was reduced to 32.5 °C for the complete virus production stage. Sabin PV type 1, 2 or 3 was added to the respective bioreactor at the defined TOI (96 or 120 h after cell inoculation) with a multiplicity of infection (MOI) of 0.0001, 0.001 or 0.01. Virus culture bioreactors were harvested after a cytopathic effect (CPE) higher than 95 % was observed.

### Harvest and clarification

After gravity-settling of the microcarriers in the bioreactor at the target CPE, harvest was collected through an internal 76 μm stainless steel sieve.

For clarification, sieved harvest was pumped (Watson Marlow 520) over two sequential filters, a 8–1 μm pore size C0HC depth filter was used, followed by a 0.5/0.22 μm pore size Express SHC filter (both from Millipore, Burlington, Massachusetts, United States) for a normal flow filtration process, at a maximum pressure cutoff (Pmax) of 0.8 bar detected by disposable pressure sensors (Pendotech).

### Concentration

Concentration was performed in two separate sequential tangential flow filtration (TFF) steps using filters with a 100 kDa MWCO (Molecular weight cutoff). The first filter was an Ultracel regenerated cellulose membrane (P2C100C01, Millipore, Burlington, Massachusetts, United States) combined with a Quattroflow 150 s (Quattroflow fluid systems, Duisburg, Germany) and disposable pressure sensors (Pendotech). The set-point for *trans*-membrane pressure (TMP) was ≤0.7 bar while pressure difference (dP) was kept below 0.5 bar*.* A back-flush procedure was implemented (≤0.3 bar) to collect all product.

The second concentration filter was a mPES hollow fibre filter (Spectrum Labs, Repligen, Breda, the Netherlands) in a presterilized disposable module (JM Bioconnect, Tilburg, The Netherlands) which consists of a 100 kDa mPES hollow fiber, mounted in a KrosFlo Research IIi TFF system (Spectrum labs; Repligen, Breda, The Netherlands). The set-point for TMP was ≤0.7 bar, while dP was kept below 0.5 bar. The set-point for feed flow was 8 mL/s and permeate flux was maintained at a maximum of 5 mL/s. The concentration factor was set at 350 and a back-flush procedure to collect all product was applied.

### Size exclusion chromatography (SEC)

SEC was performed using a column (bed height 80 ± 3 cm) containing NaOH-sanitized Sepharose CL-6B (GE Healthcare, Chicago, Illinois, United States). Column width was varied between experiments dependent on scale maintaining equal load percentage (3.5 ± 0.5 %), columns used were Vantage-L (Millipore, Burlington, Massachusetts, United States), Laboratory Research Columns (LRC) (Pall, New York, United States) or XK-column (GE Healthcare, Chicago, Illinois, United States).

### Anion exchange chromatography (AEX)

As control, DEAE-Sephadex A50 resin (GE Healthcare, Chicago, Illinois, United States) was packed into Vantage-L (Millipore, Burlington, Massachusetts, United States), Laboratory Research Columns (LRC) (Pall New York, United States) or XK-columns (GE Healthcare, Chicago, Illinois, United States) with a bed height of 20 ± 2 cm.

A convective interaction monolith (8 and 80 mL) (CiM, Bia Separations, Ajdovščina, Slovenia) with DEAE matrix was used for the purification of PV type 2 in the presence of l-arginine. For PV types 1 (In presence of l-arginine) and 3, a Poros D50 resin (DEAE) (Thermo Fisher Scientific, Waltham, Massachusetts, United States) was used.

All chromatographic experiments were conducted on an Äkta platform (GE Healthcare, Chicago, Illinois, United States). Flowrate was controlled by a dual piston pump. SEC flowrate was 3.74 cm/h, while for AEX different flowrates were used. The control Sephadex was run at 12.74 cm/h, Poros at 63 cm/h and CiM DEAE at 0.125 CV/h. UV-signals (280, 260 & 254 nm) were followed using a 2 mm flowcell for SEC and a 10 mm flowcell for AEX. Conductivity was also monitored on the Äkta platform. Results were recorded using UNICORN 6.2.3 control software (GE Healthcare, Chicago, Illinois, United States) and stored in a secure database. Different combinations of a 20 mM phosphate buffer (mixture of Na_2_HPO_4_∙2H_2_0 & KH_2_PO_4_∙H_2_0) at pH 7.0 ± 0.2 were also tested.

All PV types were purified in presence of 20 mM phosphate buffer (mixture of Na_2_HPO_4_∙2H_2_0 & KH_2_PO_4_∙H_2_0) supplemented with 50 mM l-Arginine for type 1 and 75 mM l-Arginine for type 2, no supplement was used for PV 3; all buffer pH values were set (using HCl) at pH 7.0 ± 0.2.

### Diafiltration

Diafiltration was performed with purified virus product collected from AEX using the same hardware and disposable filter setup as describe in concentration stage two. Diafiltration was started by adding a buffer consisting of M199, 5 mM phosphate and 5 g/L glycine pH 7.0 ± 0.2 in a continuous mode and a minimum of 7 times the starting volume was used.

### Inactivation

Formaldehyde inactivation was performed as described by [10]. In general, samples were first diluted to obtain a predetermined D‐antigen concentration and pH was set (7.0 ± 0.2). Inactivation was done at 37 °C for 13 days in an incubator. Complete inactivation was observed after 72–96 h as was verified by virus titration assays and inactivation kinetics.

### Process development assays

Cell numbers were determined with a Nucleocounter (Chemometec, Lillerød, Denmark). Concentrations of glucose and glutamine were measured using a Bioprofile 100 plus (Nova Biomedical, Waltham, Massachusetts, United States). Virus titre was determined by CCID50 method [22]; CPE was monitored microscopically. Yields were based on d-antigen value as measured by a fast ELISA-assay [23]. Host cell protein (HCP) impurities were determined using commercial Vero Cell HCP ELISA kit F500 (Cygnus Technologies, Southport, United States). Host-cell DNA was quantified via qPCR (, Life Technologies, resDNASEQ™ Quantitative Vero DNA Kit) using a MagnaPure system (Roche Life science, Penzberg, Germany) for DNA extraction.

### Software

MODDE (versions 9 & 12; Umetrics, Umeå, Sweden) was used to setup and analyze the design of experiments studies. Excel (Microsoft, Redmond, Washington, United States) was used for other calculations.

### Product characterization assays

These assays were performed on purified and inactivated product from the three PV types.

Animal experiments were performed according to the guidelines provided by the Dutch Animal Protection Act and were approved by the Committee of Animal Experimentation (DEC) of the National Institute of Public Health and Environment (RIVM).

IPV rat potency was performed as described in European Pharmacopoeia monograph 0214. In short, Wistar rats were immunized with different doses of ACF sIPV or the reference cIPV vaccine. After 3 weeks, serum was collected to determine the amount of specific virus neutralizing antibodies (VNT) in a CPE-based assay using Vero cells [24], [25].

The antigenic characterization of the three types in sIPV vaccine bulk product was analyzed using a surface plasmon resonance biosensor (Biacore; GE Healthcare). For each type three different monoclonal antibodies (Mabs), directed against different d-antigen epitopes*,* were used to generate an antigenic fingerprint of inactivated virus particles*.* With the biosensor, active particle concentrations - the amount of virus particles expressing the specific Mab-binding epitope - weredetermined*.*

The presence of poliovirus capsid proteins (VP1, VP2, VP3, VP4) and of HCP from Vero cells were determined with mass spectroscopy (MS). Nanoscale LC-MS was performed according to a standardized analytical procedure, using trypsin to digest proteins and ovalbumin as an internal standard [26]*.*

Stability of purified sIPV bulk product was evaluated by incubating samples for 1, 4 and 7 days at 25, 37, and 45 °C, and comparing the assay results to scores from samples stored at 4 °C. Selected assays to monitor thermal stability were d-antigen ELISA, and the active virus particle concentration as determined with biosensor analysis in the Biacore.

## Results

**Fig. 1:**
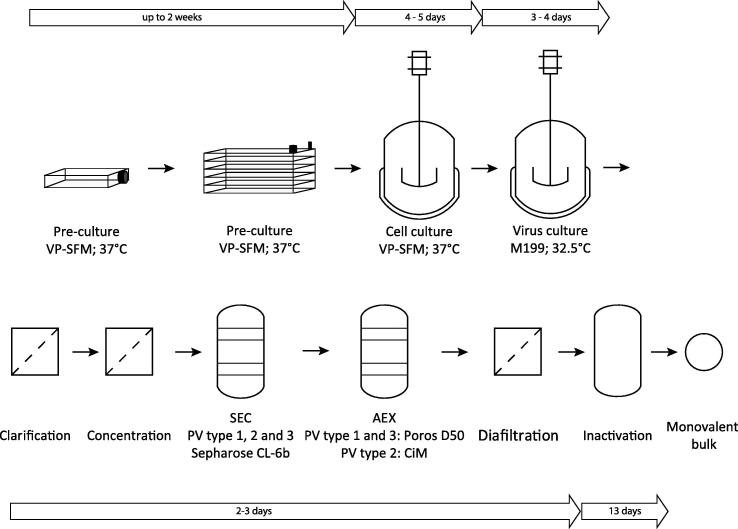
Flow diagram of the ACF production process of sIPV monovalent bulk. Pre-culture of cells was done in *T*-flasks and cell factories prior to start of the cell culture in bioreactors. After media exchange virus culture was started. Virus was harvested, clarified and concentrated prior to purification with two chromatography steps. For different viruses different resins were used. The diafiltration step prior to inactivation was performed for PV type 1 and 2. The associated buffer exchange was not required for PV type 3. Inactivation with formaldehyde was the final step to generate a monovalent bulk (indicated bulk circle). Trivalent sIPV was prepared by mixing inactivated monovalent bulks of PV 1, 2 and 3. A diagram for cIPV can be found in Thomassen et al. (2013) [25].

The development described here is aimed at removing all animal compounds from the sIPV process. Doing this, resulted in almost the same train of unit operations (Fig. 1) as in the base case sIPV and cIPV processes. However, before product inactivation, diafiltration was included as means to remove the arginine added prior to the chromatographic steps. Finally, the overall process was checked for robustness using a DoE approach. These results will be further discussed in the following sections.

### Process development (Sabin poliovirus type 2) – upstream process

Two material changes were implemented in order to turn Vero cell growth and virus production into an ACF process. The first one, was the change from SC medium to VP-SFM. Results showed that Vero cells could grow normally, and that virus production was increased. The second change was the switch from a porcine source trypsin into an ACF protease (TrypLE™ Select, Thermo Fisher) for cell detachment. Doing this had no apparent effects on cell growth nor efficiency in cell detachment. In addition, use of antibiotics was excluded from the ACF process.

### Vero cell expansion

Transfer of Vero cells from a SC cell bank to VP-SFM medium was performed as a single-step without notable changes in growth characteristics such as growth rate, viability or cell morphology. Data from 30 vial thaws showed that the first step overall recovery was 73 %, while initial viability of the frozen cell suspension was 92 %. Moreover, the mean growth rate of Vero cells was 0.022 h^−1^, which increased slightly to 0.024 h^−1^ in the second passage. The increase in the amount of viable cells seeded in a new flask and the amount of cells harvested after enzymatic detachment for the next passage, showed that VP-SFM medium was able to support more than three population doublings of Vero cells per passage.

### Cell growth in bioreactors

**Fig. 2:**
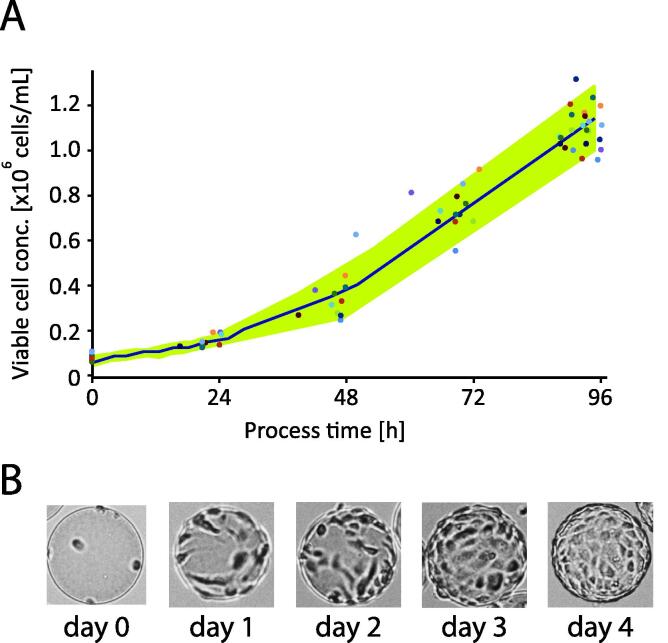
Growth of Vero cells adherent to Cytodex 1 microcarriers (3 g/L) in VP-SFM during the first 96 h after inoculation at 10L scale in 15L bioreactors Panel A: Mean growth curve is given in blue, standard variation in green (n = 12). Points represent measurements of individual bioreactors. Panel B: photos showing representive pictures of Vero cells on microcarriers during the cell growth. (For interpretation of the references to colour in this figure legend, the reader is referred to the web version of this article.)

In lab scale bioreactors, Vero cells in VP-SFM medium grew from a seeding density of 0.1 × 10^6^ cells/mL up to densities of 1.2–1.3 x10^6^ cells/mL after 96 h, and to 1.5–1.65 x 10^6^ cells/mL after 120 h. The average measured growth rates were 0.024–0.25 h^−1^ and 0.019–0.020 h^−1^, respectively. The decrease in the latter occured because 96 h is at the end of the exponential growth phase, where cells are reaching confluency on microcarriers (Fig. 2 panel B). Fig. 2 shows the growth curve of twelve 15L bioreactor runs until virus inoculation (TOI = 96 h). It can be observed that Vero cells growing in VP-SFM show a stable, reproducible performance.

### Virus production in bioreactors

The cIPV production process uses serum containing media (EMEM + 5 % bovine serum) for cell growth and the ACF M199 media for virus production. An initial attempt for a ACF alternative used VP-SFM for both phases. However, this resulted in formation of aggregates causing blocking of the concentration UF and HF filters. Because of this, it was decided to use VP-SFM for cell growth and M199 for virus production. This not only resulted in a process more comparable to the cIPV but also considerably decreased the formation of aggregates.

Infection with Sabin PV type 2 after 96 h of cell culture (TOI) with a MOI of 0.001 resulted in a d-antigen concentration in crude harvest of approximately 48 ± 7 DU/mL (n = 12). These values are roughly twice as high as those previously found using SC EMEM and M199 medium (20–25 DU/mL at different scales) [11]. Moreover, variation in MOIs (0.01, 0.001, and 0.0001) or TOIs (96 and 120 h) did not result in any significant differences in d-antigen concentration in crude harvest.

## Process development (Sabin poliovirus type 2) – downstream process

### Harvest and clarification

No issues were observed when performing harvesting of virus culture from an ACF USP process. Hence, the method was not further studied. Similarly for clarification, which was performed to remove cellular debris. This operation, also did not require any substantial changes with respect to equipment or process parameters (PPs) associated with the shift from SC to ACF medium.

### Concentration

Concentration was performed as a two-step process, using two TFF filters with 100kD filter MWCO. The second filter was included to reduce the internal holdup volume and reach a final concentration factor of 325 ± 25, thereby facilitating the rinse/back-flush procedure and increase d-antigen recoveries.

To make this operation ACF, it was necessary to change the filter cleaning procedure, because porcine trypsin was previously used. It was replaced with sodium hydroxide (NaOH). However, doing so resulted in a loss of filter integrity of the used poly-sulfone (PTHK) filters. Therefore, this filter was replaced by a regenerated cellulose membrane (PLCHK, 100kD) that could be cleaned with NaOH up to 25 cycles without any negative effects.

To optimize this operation, it was determined that for a feasible TFF performance the clarified harvest should have a maximum turbidity of 0.9 NTU. The TMP was maintained below 0.7 bar for both steps. The maximum permeate flux for the second step was determined as of 5 mL/s. A maximum concentration factor of 350 times was identified to be able to withstand the potential variations in clarified ACF harvest and to be compatible with standard operation of the following chromatography steps.

By implementation of these modifications and by applying a back-flush procedure, the combined yield of this step was higher than 95 %. To conclude, under ACF conditions formation of a gel layer on tangential membrane was difficult to prevent. However, this does did not cause irreversible changes to the product. In addition, the use of the second smaller 100kD filter facilitated harvest of this material in the flush procedure.

### Purification with SEC and AEX

In development of a sIPV process based on the cIPV process, severe aggregation of PV type 2 was found during column chromatography because the isoelectric point (pI) of Sabin PV type 2 was close to that of the buffer used for SEC/AEX. Though the number of mutations between Salk PV and Sabin PV strains are limited, the surface chemistry of formed virus particles is sufficiently different to create considerable differences in pI [12]. For Sabin PV type 2, this meant that the pI came close to the pH of the standard buffer used for purification of PV with size-exclusion and ion-exchange chromatography. This caused the virus product to aggregate and resulted in a low yield for this initial base case process. Common options to prevent this, like a shift in pH or addition of salt, did not support a feasible improved process. It was also preferred to use the same elution buffers for SEC and AEX so an intermediate diafiltration step to exchange buffers could be prevented. During testing of several components, it was shown that addition of the basic amino acid l-arginine to the buffer considerably reduced aggregation of virus product [27] and the overall DSP yield increased from near 5 % to nearly 40 %.

**Fig. 3:**
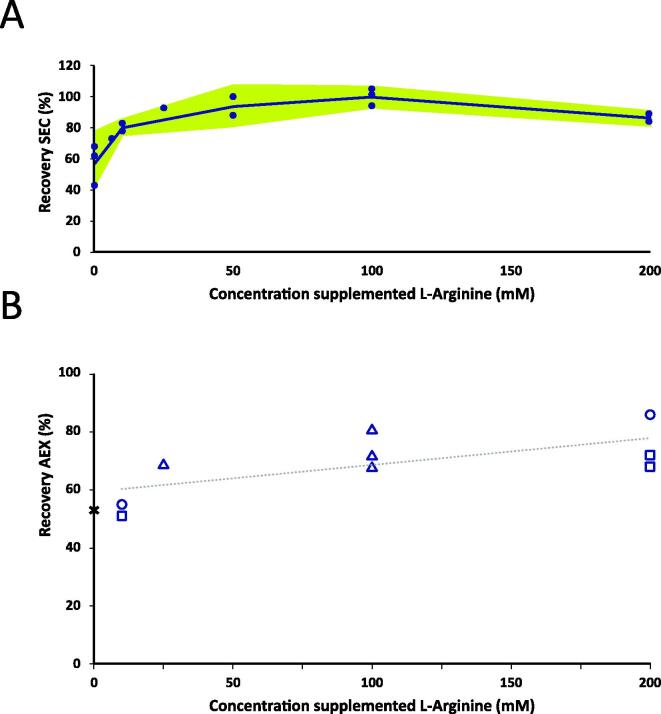
Results optimization chromatography: Panel A l-arginine increased the recovery of sIPV type 2 during SEC (mean with 95% confidence interval) dots represents individual measurements and Panel B: AEX l-arginine increased recovery of AEX. Circles, triangles and squares represent corresponding material with concentration factors of 500, 750 and 1000-fold (reference is partially-optimized control; for reference base case sIPV AEX recovery was 32% (Thomassen 2013, Plos one)).

In this project, the use of l-arginine was selected, and concentration ranges to support improved performance were checked. Conditions for SEC were based on previously established process, meaning that resin type, bed height and flows were kept constant, comparable to that for cIPV*.* The effect of l-arginine in this step became visible at concentrations above 10 mM, while the optimum range for SEC appeared to be between 50 and 100 mM (Fig. 3a). Under these conditions, with small variations in concentration of l-arginine, the yield of Sabin PV type 2 for SEC chromatography step was in the range from 80 to 100 %.

The AEX column step required a general improvement. This involved replacement of DEAE based resin, mainly because it was a single-use material with limited scalability. A resin screening was done to select appropriate replacement. For PV type 1 and 3 a Poros D50 resin was chosen. For PV type 2, a CIM DEAE resin was selected, which supported a feasible performance at a revised flow rate of 0.125 CV/min, using a radial flow column. Optimum concentrations range of l-arginine for AEX application for Sabin PV type 2 was determined at 25–170 mM. This has sufficient overlap with the concentration range for SEC. Therefore, the same elution buffer could be used for both column steps. Under these conditions, the AEX step yield for Sabin PV type 2 was above 65 % (Fig. 3b). This step yield was relatively independent from the concentration factor (CF) tested during the concentration step screening (CF 500, 750, and 1000).

### Diafiltration

Presence of higher concentrations of l-arginine in the elution buffer of AEX has an inhibiting effect on inactivation efficiency of formaldehyde [28]. Therefore, a diafiltration step was introduced (100kD TFF filter) to reduce the concentration of l-arginine to concentrations that do not interfere with product inactivation. This diafiltration was performed without (measurable) loss of product*.*

### Process robustness

To test performance of the improved overall purification process, and to check its robustness to set-point variations, a DoE study was performed (Fractional factorial resolution 3, 2^(7−4)^). Process parameters (PPs) to be included in this study (Table 1) were selected based on results of a risk assessment [29], which evaluated the possibility of each PP to affect the selected critical quality attributes (CQAs) (d-antigen yield and HC-DNA and HCP concentrations) measured after the AEX-step. The CQAs were measured after this step because, as mentioned above, dialfiltration and inactivation were not part of the optimization studies.

**Table 1:** List of parameters tested on the DoE for the robustness study. Parameters were ranked based on expected relative importance to affect product yield. Number 1 is expected to be the most critical.

Ranking	Parameter	Unit operation	Units	Center point	Target value	High value	Low value
1	Arginine concentration	SEC and AEX	mM	70	75	90	50
2	Product load	SEC	%	3.75	3.50	5.00	2.50
3	pH buffer	SEC and AEX	–	7.0	7.0	7.2	6.8
4	Concentration factor	Concentration	–	310	350	350	270
5	TMP	1st concentration step	bar	0.58	0.45	0.70	0.45
6	TMP	2nd concentration step	bar	0.58	0.45	0.70	0.45
7	Flowrate	Clarification	L/h	4.5	3.3	6.5	2.5

Hence, ten bioreactor runs at 10L working volume scale were performed at set-point to generate sufficient harvest for DSP. Every PP which was not tested in the DoE was kept at set-point throughout all runs. Overall DSP yields, not including inactivation, for these runs ranged from 49 % to 85 %. The standard above described ACF DSP process scored a 65 % recovery. For the main contaminants, it was found that HC-DNA was below specified limit for all purified fractions and that HCP was ranging from less than 0.08 (detection limit) to 6.81 mg/L. One of the fractions was slightly above the specification of 6.25 mg/L of HCP for the final product, however the following diafiltration step, meant to reduce the concentration of l-arginine, resulted in a further reduction of HCP from 6.81 μg/mL to 0.18 μg/mL.

Finally, diafiltration and inactivation were performed using the target sIPV ACF process (run at defined set-points). This resulted in an overall product recovery of 37 % after inactivation and impurity concentrations below required specifications. These results show that the target DSP for ACF sIPV type 2 had a performance that may be expected for a commercial IPV process. The small scale DSP also had sufficient robustness as indicated by the finding that all applied variations in process parameters resulted in a feasible sIPV product yield (>49 % after AEX) with an acceptable impurity profile (below specification limits).

### Product characterization (all three Sabin PV types)

Product obtained after processing all three Sabin PV types was characterized. Their potency was evaluated by performing the standard rat potency test [25], [30] and the products proteins and impurity levels were evaluated. In addition, the stability of the product was evaluated at different temperatures.

For Sabin PV type 2, the production process was run according to the settings of the optimization development performed for this strain, while for Sabin PV type 1 and 3 previously established settings were used. Vaccine product characteristics were compared to SC-produced sIPV [10] or cIPV [11].

### Potency (immunogenicity)

**Fig. 4:**
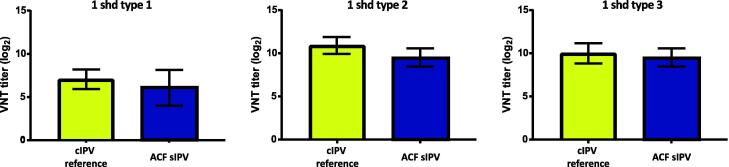
Comparison of virus neutralizing antibodies in a single human dose of cIPV (reference) and sIPV ACF for PV type 1, 2 and 3. Values are expressed as mean ± standard deviation (n = 10 animals).

Measured VNT levels showed that a single human dose (SHD) of ACF sIPV induced similar maximum VNT as the reference cIPV vaccine (Fig. 4), and that its immunogenicity was comparable to that of sIPV vaccine derived from the SC-production process [10].

### Antigenic characterization

**Fig. 5:**
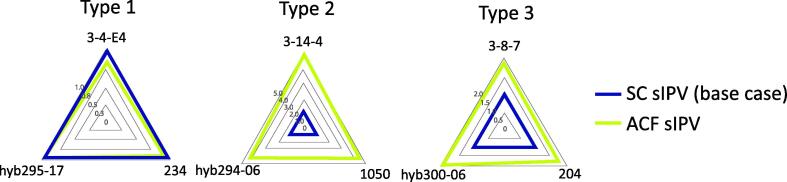
Comparison of d-antigenic fingerprint of purified monovalent sIPV products from ACF (green) and SC (blue) process. Values represent d-antigen concentrations determined with Biacore using three different monoclonal antibodies per PV type (PV type 1 antibodies 3-4-E4; hyb295-17 and 234; PV type 2 antibodies 3-14-4; hyb294-06 and 1050; PV type 3 antibodies 3-8-7; hyb300-06; 204). Grey line indicate axis values. (For interpretation of the references to colour in this figure legend, the reader is referred to the web version of this article.)

Antigenic fingerprinting can be used to illustrate vaccine quality and was previously used to compare sIPV to cIPV [31]. We have used antigenic fingerprinting to assess quality differences between SC-produced sIPV and ACF-derived sIPV products. For each PV type three different monoclonal antibodies (Mabs), directed against different d-antigen epitopes, were used to determine the amount of active particles with a biosensor (Biacore). While concentrations differ between the products no significant differences between the immunochemical properties were observed, indicated by the parallelity of the lines (Fig. 5). The specific d-antigen antigenicity and activity per amount of protein for sIPV product from the ACF-process was slightly higher than for the SC-produced sIPV product for sIPV type 1 and 3. For ACF sIPV type 2 product, the specific antigenicity was 2–3 times higher compared to the SC sIPV vaccine.

### Protein analysis

MS results indicated that the PV VP1-VP4 capsid proteins were most dominant in the purified bulk product fractions (data not shown). Concentrations of the Vero cell-derived HCP were very low in all samples, confirming results from the Vero cell HCP ELISA.

### Thermostability

The (accelerated) stability test of ACF sIPV type 1, 2, and 3 showed that product was stable with respect to d-antigen and active virus particle concentration during 7 days at 25 and 37 °C, but that some d-antigen configuration was lost during the 1-day incubation at 45 °C (data not shown). The findings are in line with results from a previous thermostability study of sIPV [32].

## Discussion

The sIPV process was adapted to be completely free of animal components. Besides a change in culture medium from serum-containing to an ACF medium, the porcine trypsin used for detachment of adherent Vero cells and for cleaning of ultrafiltration (UF) membranes in the purification process had to be replaced by ACF variants. Replacement of porcine trypsine with ACF alternatives TrypLE Select for Vero cell detachment and NaOH for UF cleaning required no additional process modifications. In addition, cell growth of Vero cells in VP-SFM medium was comparable to growth in SC medium. However, crude harvest from virus producing cultivation process in VP-SFM medium, caused major product aggregation in the primary recovery steps (clarification and concentration). Product aggregation during primary recovery was prevented by the use of M199 medium during virus culture and by limiting the concentration factor during TFF concentration step to maximum 350×. It is not exactly known what the major factors are that contributed to the better performance of M199 regarding primary recovery. A contribution might be found in reduction of fibronectin. Medium replacement can reduce the concentration of fibronection which is a promoting factor of aggregation [33]. In addition, M199 medium contains components such as Tween that will support solubility of proteins.

A considerable higher product yield, when compared to the serum containing sIPV process, was achieved during implementation of ACF. The ACF USP process, provided a nearly twofold higher yield of virus in harvest compared to the previously used SC USP process, determined both by virus titration (CCID50) and by d-antigen (ELISA). For Sabin PV type 2, the d-antigen yield was 25 DU/mL in SC medium, while 48 DU/mL was determined for the runs with the ACF combination of VP-SFM and M199 medium. For Sabin PV type 1 and 3, a similar improved performance of the sIPV USP production process with ACF media was established. This higher PV production yield was attributed to a higher specific PV production rate. This increase in specific production rate was caused by cell growth on VP-SFM as other factors such as cultivation temperature remained the same [34].

Optimization of the purification process was essential as in the base case process [10] the overall DSP yield for Sabin PV type 2 was only 5 % due to virus aggregation. As the common options to prevent virus self-aggregation, like a shift in pH or addition of salt, did not support a feasible improved process. It was chosen to add the basic amino acid l-arginine to the buffer which has been demonstrated to considerably reduce aggregation of virus product [27]. Indeed, addition of l-arginine to the elution buffers in chromatography resulted in an overall DSP yield of 40 %. With the adjustments of the purification process to an ACF process, an optimization of the production yield was achieved. This increase will most probably result in a decrease of the estimated cost of goods. An analysis of cost of goods for sIPV has predicted sIPV manufacturing to be cost-competitive when USP yield would increase at least 1.5-fold and DSP recovery would approximate DSP recoveries comparable to cIPV (approximately 40 % [11]) [7].

A DoE study with variation in the DSP process has been done to evaluate process robustness. Results show that the target DSP for ACF sIPV type 2 performed as expected for a commercial IPV process.

Evaluation of a production process by assessing impacts of changes in a process parameter not just within a unit operation but also beyond is a challenge for process developers as process have several successive unit operations and a certain number of parameters per unit operation. Studying variation of all PPs would results in a large amount of experiments at a representative scale. The approach followed here, to limit the amount of PP based on risk assessment allowed to limit the DoE study to 10 bioreactor runs at 10L scale. This illustrates it is feasible to study multi-unit operation processes in such way.

In addition, such a QbD approach allows understanding of impact of potential variations occurring during manufacturing.

## Conclusions

An ACF sIPV process has been developed that has a competitive efficiency and an improved overall quality. The development work has been mainly performed for Sabin PV type 2, which was the virus type that required most improvement in efficiency and yield. Nevertheless, the developed standard pilot process also indicated appropriate feasibility for Sabin PV type 1 and 3. The product quality properties for all three virus types included in sIPV vaccines were comparable to the standards of cIPV.

## Declaration of Competing Interest

The authors declare that they have no known competing financial interests or personal relationships that could have appeared to influence the work reported in this paper.

## Data Availability

The data that has been used is confidential.
